# Critical role of reactive oxygen species (ROS) for synergistic enhancement of apoptosis by vemurafenib and the potassium channel inhibitor TRAM-34 in melanoma cells

**DOI:** 10.1038/cddis.2017.6

**Published:** 2017-02-02

**Authors:** Daniel Bauer, Felix Werth, Ha An Nguyen, Felix Kiecker, Jürgen Eberle

**Affiliations:** 1 Department of Dermatology, Venerology und Allergology, Skin Cancer Center Charité, Charité – Universitätsmedizin Berlin, Berlin, Germany; 2 Molecular Medicine Master's Program, Charité – Universitätsmedizin Berlin, Berlin, Germany; 3 Institute for Biochemistry and Biology, Faculty of Science, University of Potsdam, Potsdam, Germany

## Abstract

Inhibition of MAP kinase pathways by selective BRAF inhibitors, such as vemurafenib and dabrafenib, have evolved as key therapies of BRAF-mutated melanoma. However, tumor relapse and therapy resistance have remained as major problems, which may be addressed by combination with other pathway inhibitors. Here we identified the potassium channel inhibitor TRAM-34 as highly effective in combination with vemurafenib. Thus apoptosis was significantly enhanced and cell viability was decreased. The combination vemurafenib/TRAM-34 was also effective in vemurafenib-resistant cells, suggesting that acquired resistance may be overcome. Vemurafenib decreased ERK phosphorylation, suppressed antiapoptotic Mcl-1 and enhanced proapoptotic Puma and Bim. The combination resulted in enhancement of proapoptotic pathways as caspase-3 and loss of mitochondrial membrane potential. Indicating a special mechanism of vemurafenib-induced apoptosis, we found strong enhancement of intracellular ROS levels already at 1 h of treatment. The critical role of ROS was demonstrated by the antioxidant vitamin E (*α*-tocopherol), which decreased intracellular ROS as well as apoptosis. Also caspase activation and loss of mitochondrial membrane potential were suppressed, proving ROS as an upstream effect. Thus ROS represents an initial and independent apoptosis pathway in melanoma cells that is of particular importance for vemurafenib and its combination with TRAM-34.

For decades, melanoma remained a deadly disease lacking an effective therapy in the metastatic stage. The situation changed with the identification of activating BRAF mutations in about one-half of cutaneous melanomas.^1^ Selective BRAF inhibitors (vemurafenib, dabrafenib) as well as inhibitors of the downstream MAP kinase MEK (trametinib, cobimetinib) have now been approved for therapy of BRAF-mutated melanomas.^2, 3^ Especially, combinations of BRAF and MEK inhibitors have shown substantial benefit for patients with regard to significant prolongation of overall survival.^4, 5^ The situation may even further improve with combinations of immune checkpoint inhibitors, such as anti-CTLA4 and anti-PD1.^6^ Nevertheless, tumor relapse and therapy resistance are still frequent and often follow within only a few months.^7^ Thus, although present clinical results are highly encouraging, further improvements and particularly new, even more effective combinations will be needed to finally overcome melanoma mortality.

Different cellular mechanisms may contribute to therapy resistance of cancer,^8^ of which apoptosis deficiency may be considered as the major cause. This is explained by the fact that elimination of cancer cells through proapoptotic programs represents the common end path of most anticancer strategies. For example, different chemotherapeutic drugs cause cellular or DNA damage, which induces cell-intrinsic proapoptotic pathways; and also BRAF inhibitors induce apoptosis or sensitize for proapoptotic programs.^9, 10, 11^

Two major apoptosis pathways have been described that are initiated by the interaction of death ligands with death receptors (extrinsic pathway) and by cellular or DNA damage (intrinsic pathway), respectively. Downstream, caspase cascades are activated consisting of initiator and effector caspases, such as caspase-3.^12^ In several tumor cells, including melanoma cells, efficient apoptosis induction needs proapoptotic mitochondrial activation.^9, 13^ Mitochondrial contribution to apoptosis is closely related to a loss of mitochondrial membrane potential (MMP) and release of proapoptotic, mitochondrial factors. This pathway is critically controlled by the family of proapoptotic and antiapoptotic Bcl-2 proteins.^14^

Besides the well-established pathways, there is increasing evidence for alternative and possibly supplementary pathways. Thus reactive oxygen species (ROS) may initiate independent apoptosis programs.^15, 16^ In melanoma cells, we have previously found enhanced ROS levels in response to an iron-containing nucleoside analog and to the phospoinositol-3 kinase inhibitor wortmannin.^17, 18^ Elevation of ROS was proven as critical for apoptosis induction, as apoptosis was prevented by the ROS scavenger *α*-tocopherol.

Membrane ion channels, which serve fundamental cellular functions, represent additional, promising tumor targets. Thus Ca^2+^-dependent potassium channels contribute to cytoplasma membrane hyperpolarization facilitating Ca^2+^ entry, a prerequisite for cell proliferation. Overexpression of the family member KCa3.1 (IK1) was related to aberrant cell proliferation of different tumor cells, including melanoma.^19, 20^ TRAM-34 has been established as a selective KCa3.1 inhibitor, which avoids side effects of some other channel inhibitors, such as hepatotoxicity.^21, 22^ We have previously shown that TRAM-34 can act as apoptosis enhancer in melanoma cells when combined with the death ligand TRAIL (TNF-related apoptosis-inducing ligand).^23^ Here we demonstrate that apoptosis induction by vemurafenib in melanoma cells is strongly enhanced by TRAM-34. Unraveling the pathway identified ROS generation as a general factor in vemurafenib-induced apoptosis, which appears of particular importance for the combination with TRAM-34.

## Results

**Figure 1:**
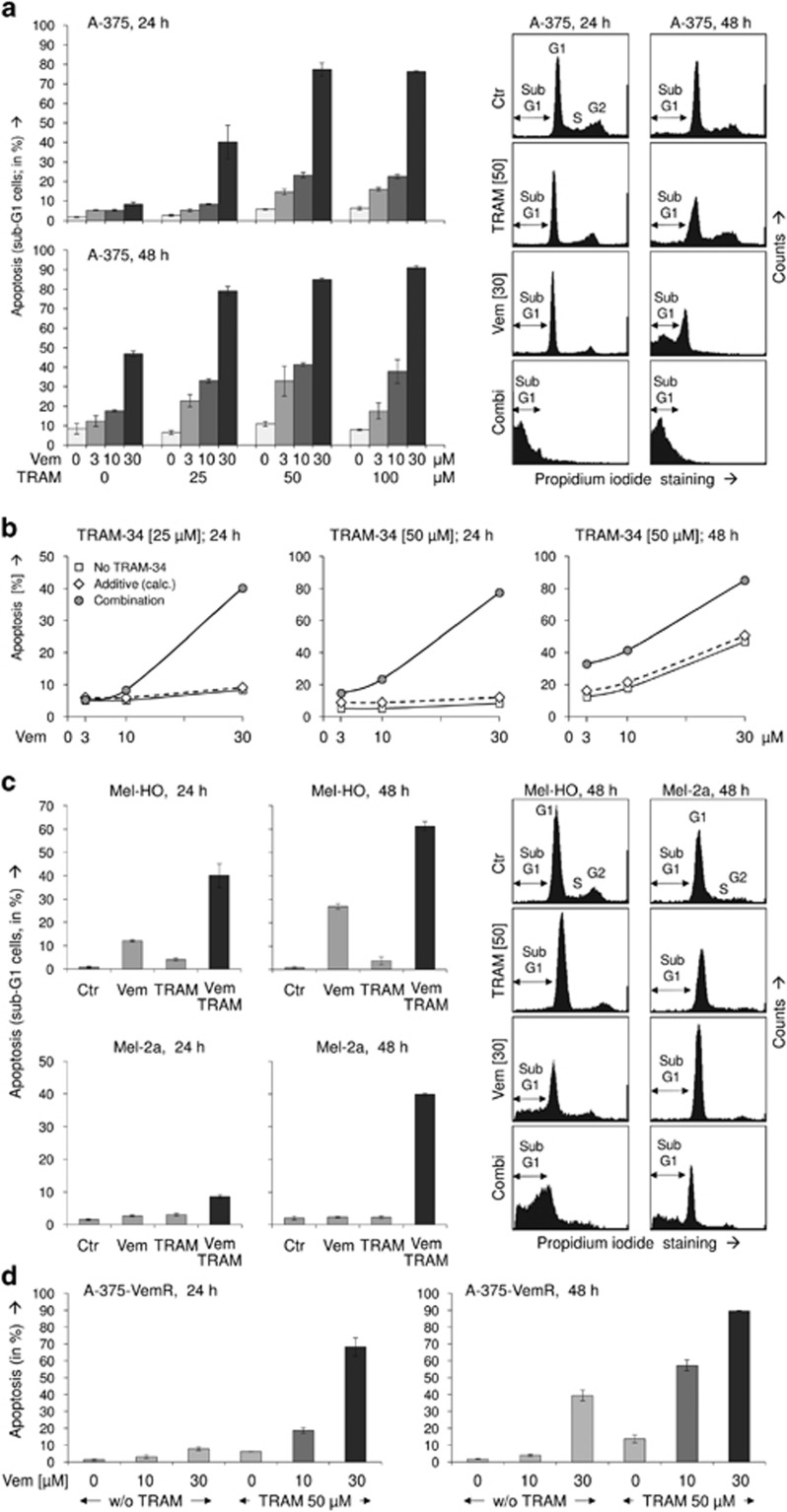
(**a**) Apoptosis induction is shown in the melanoma cell line A-375 at 24 and 48 h of treatment with vemurafenib (Vem; 3, 10, 30 *μ*M) and TRAM-34 (TRAM; 25, 50, 100 *μ*M). Apoptotic effects with the concentrations of 30 *μ*M Vem and 50 *μ*M TRAM, used for most subsequent experiments, have been reproduced multiple times (>3 × ). Apoptosis values correspond to cells with a less DNA content than cells in G1 phase, which is due to DNA fragmentation (sub-G1). Examples are given on the right side. Cell populations in G1, G2 and S-phase as well as sub-G1 cells are indicated. (**b**) For three conditions (24 h, TRAM-24, 25 *μ*M and 50 *μ*M; 48 h, 50 *μ*M TRAM-24), calculated additive effects on apoptosis (diamond symbols) are directly compared with experimentally determined combination effects (circle symbols, corresponding to panel (**a**)). Calculated additive effects result from direct addition of apoptosis by vemurafenib and apoptosis by TRAM-34. For further comparison, apoptosis by vemurafenib alone (3, 10, 30 *μ*M) is shown (square symbols). (**c**) Melanoma cell lines Mel-HO and Mel-2a were treated with selected concentrations of Vem (30 *μ*M) and TRAM (50 *μ*M). (**d**) Combinations of TRAM (50 *μ*M) and Vem (10, 30 *μ*M) were used for treatment of an A-375 cell population selected for vemurafenib resistance. All experiments have been performed with triplicate values, and at least two independent experiments for panels (**c** and **d**) revealed highly comparable effects

For identifying suitable combination partners, which may enhance the antitumor activities of vemurafenib, a screening with several pathway inhibitors and apoptosis agonists was performed (data not shown). Synergistic enhancement of apoptosis in the combination of vemurafenib and TRAIL, which had been described previously,^11^ served as positive control. Of the different effectors tested, TRAM-34, an inhibitor of the potassium channel KCa3.1, appeared of particular value. Although it did not trigger apoptosis by itself, TRAM-34 strongly enhanced and accelerated vemurafenib-induced proapoptotic effects. Vemurafenib was applied in a standard concentration of 30 *μ*M as well as in two reduced concentrations of 3 and 10 *μ*M. TRAM-34 further enhanced apoptosis at all tested concentrations. Thus apoptotic rates were stepwise increased when raising the concentration of TRAM-34 from 0 to 25 to 50 *μ*M. For example, at 48 h, the numbers of apoptotic cells increased from 12% to 23% to 33% for 3 *μ*M vemurafenib, from 18% to 33% to 41% for 10 *μ*M vemurafenib and from 49% to 79% to 86% for 30 *μ*M vemurafenib (Figure 1a). These effects were clearly more than additive, particularly as there was only limited proapoptotic effect of TRAM-34 alone (Figure 1b).

In order to generalize the findings on enhanced apoptosis, two more melanoma cell lines (Mel-HO and Mel-2a) were investigated. All three cell lines carry BRAF mutations, but whereas A-375 and Mel-HO were responsive to vemurafenib single treatment, Mel-2a revealed pronounced resistance. Nevertheless, comparable enhancement of apoptosis was seen in all three melanoma cell lines for the combination of 30 *μ*M vemurafenib+50 *μ*M TRAM-34 *versus* vemurafenib alone. Thus, in Mel-HO, apoptosis rates increased at 48 h from 27% to 62% by the combination with TRAM-34. In vemurafenib-resistant Mel-2a, apoptosis rates raised from <5% at 48 h up to 40±2% (Figure 1c). This suggests that the combination effect reported here may be representative for melanoma cells, even in case of intrinsic vemurafenib resistance.

To further prove the issue of vemurafenib resistance, another model was established in A-375. Cells were cultured with successively increasing concentrations of vemurafenib up to 10 *μ*M within a period of 4 weeks. The selected cells showed continuous growth despite vemurafenib and revealed no apoptotic response to 10 *μ*M. Nevertheless, these cells were significantly responsive to the combined treatment of vemurafenib/TRAM-34. In response to 10 *μ*M vemurafenib+TRAM-34, apoptosis was induced up to 19% and 58% at 24 and 48 h, respectively. Similarly, apoptosis induced by 30 *μ*M vemurafenib was enhanced (Figure 1d). Thus both intrinsic resistance (Mel-2a) and induced vemurafenib resistance (A-375-VemR) were overcome in these models.

**Figure 2:**
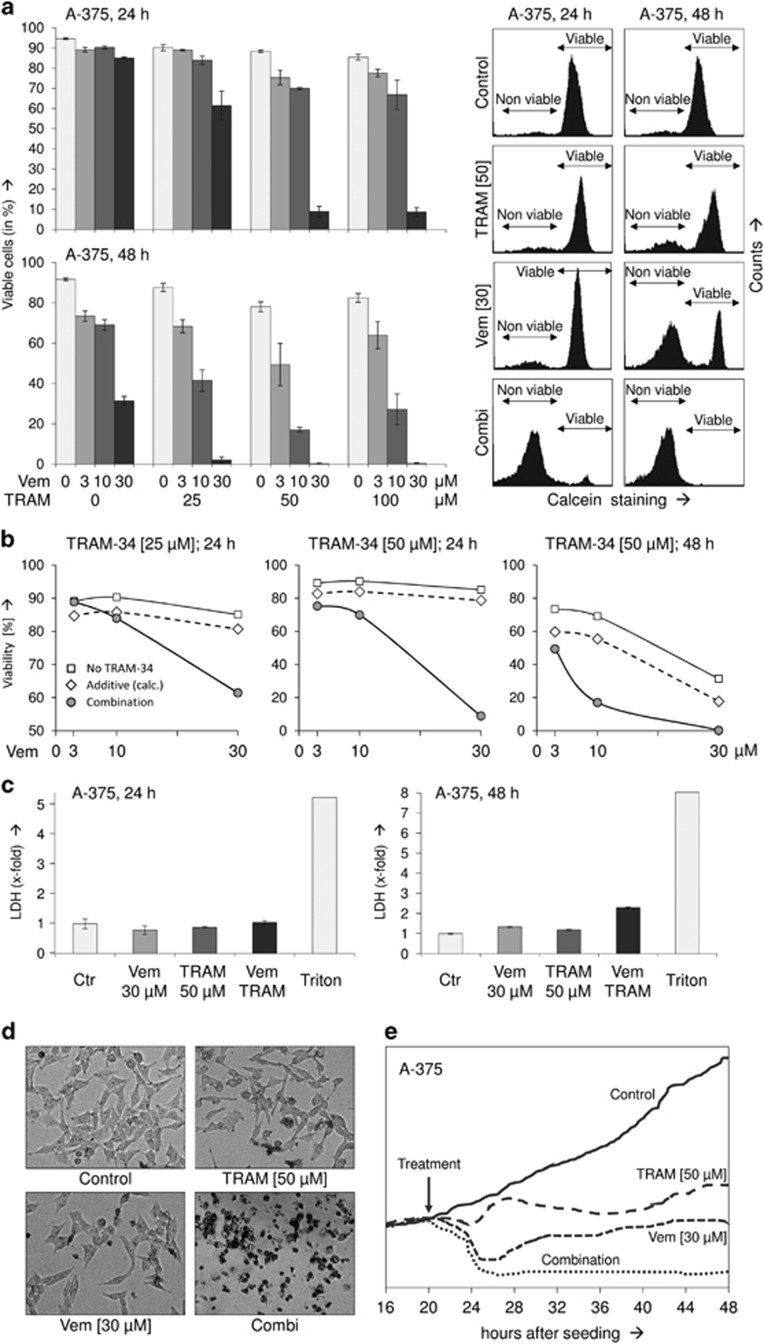
(**a**) Cell viability was determined by calcein staining in A-375 cells at 24 and 48 h in response to vemurafenib (Vem; 3, 10, 30 *μ*M) and TRAM-34 (TRAM; 25, 50, 100 *μ*M). Effects on cell viability with the selected concentrations of 30 *μ*M Vem and 50 *μ*M TRAM have been reproduced at least two times. Examples of calcein-stained cells are given on the right side. Non-viable and viable cell populations are indicated. (**b**) For three conditions (24 h, TRAM-34 25 *μ*M and 50 *μ*M; 48 h, TRAM-24 50 *μ*M), calculated additive effects on viability (diamond symbols) are directly compared with experimentally determined combination effects (circle symbols, corresponding to panel (**a**)). Calculated additive effects result from addition of the negative effects by vemurafenib and TRAM-34. For further comparison, effects by vemurafenib alone (3, 10, 30 *μ*M) is shown (square symbols). (**c**) Relative cytotoxicity was determined by quantification of released LDH. Cells completely lysed by triton-x100 treatment (Triton) served as positive controls (triplicate values, >2 independent experiments). (**d**) Rounded and detached cells were characteristic of combination treatment at 24 h. (**e**) Adherent cells corresponding to cell proliferation were determined in real time by the xCELLigence system (triplicate values). Time of treatment is indicated by an arrow

Enhanced apoptosis went along with a reciprocal loss of cell viability, as determined by calcein staining and subsequent flow cytometry. At all vemurafenib concentrations used (3, 10, 30 *μ*M), TRAM-34 further decreased cell viability. Thus, at 48 h, the numbers of viable cells in response to vemurafenib stepwise further decreased when raising TRAM-34 concentration from 0 to 25 to 50 *μ*M. Values decreased from 73% to 68% to 39% (Vem 3 *μ*M), from 69% to 42% to 17% (Vem 10 *μ*M) and from 31% to 2% to 0.5% (Vem 30 *μ*M) (Figure 2a). Also concerning cell viability, the combination effects were far more than additive (Figure 2b).

In order to distinguish between direct cytotoxic effects (cell lysis) and apoptosis induction, release of lactate dehydrogenase (LDH) into the cell culture supernatant was quantified by an enzymatic assay at 24 and 48 h. In contrast to induced apoptosis, LDH activity in cell culture supernatant remained at a low level, thus indicating that cells were not lysed up to 24 h. Only at 48 h of combination treatment, LDH levels were slightly enhanced by 2.3-fold as compared to control cells, which may be explained by secondary cell lysis following apoptosis in cell culture (Figure 2c). Complete cell rounding and detachment, characteristic of apoptotic cells, were seen already at 24 h of combination treatment (Figure 2d), and monitoring attachment of cells in real time (xCELLigence system) confirmed that both single treatments resulted in only intermediate effects, while the combination of TRAM-34 and vemurafenib resulted in complete cell detachment (Figure 2e).

**Figure 3:**
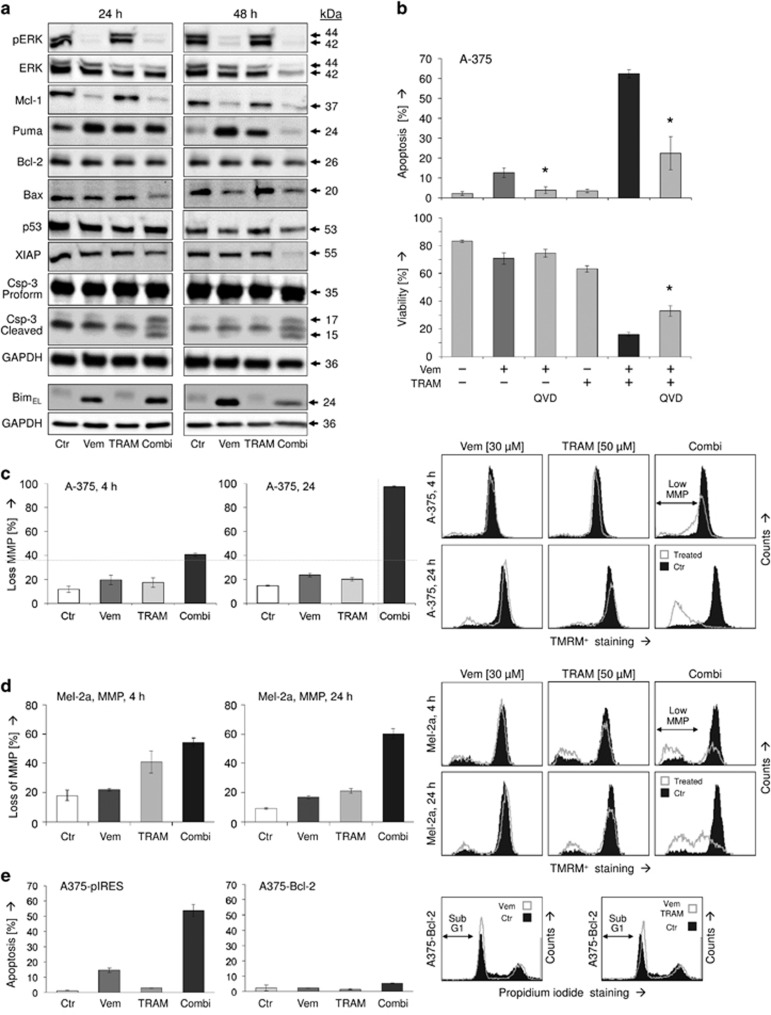
(**a**) Protein expression was determined by Western blotting in A-375 cells at 24 and 48 h in response to treatment with 30 *μ*M vemurafenib (Vem), 50 *μ*M TRAM-34 (TRAM) and the combination (Combi). Expression was compared with non-treated cells (Ctr). Proteins analyzed: phosphorylated ERK (pERK), total ERK (ERK), antiapoptotic factors (Mcl-1, Bcl-2, XIAP), proapoptotic factors (Puma, Bim_EL_, Bax, p53), and caspase-3 (proform, 35 kDa; active, cleaved forms, 15 and 17 kDa). Each determination was repeated with a second independent series of protein extracts, which showed highly comparable results. (**b**) The significance of caspase activation was proven by additional treatment with the pan-caspase inhibitor QVD-OPh (QVD, 10 *μ*M). (**c** and **d**) Loss of MMP was determined by the potential-sensitive dye TMRM^+^ at 4 and 24 h of treatment in A-375 and Mel-2a. Examples, as determined by flow cytometry, are shown in the right panels. Cells with low MMP are indicated. (**e**) Apoptosis was determined in a Bcl-2-overexpressing A-375 cell clone (A375-Bcl-2). It was compared with a sensitive mock/plasmid-transfected A-375 cell clone (A375-pIRES). (**b**–**d**) All determinations were carried out in triplicates; asterisks indicate statistical significance

Thus apoptosis appeared as the dominant effect of the combination vemurafenib/TRAM-34 demanding an unravelling of the pathways involved. As expected, vemurafenib almost completely abolished ERK phosphorylation. Total ERK was also downregulated at 48 h of combination treatment, which may be explained by strong apoptosis induction at this time. In contrast, TRAM-34 remained without effect on ERK. For 30 *μ*M vemurafenib applied, only little rebound ERK activation was seen at 48 h, indicating a largely sustained effect (Figure 3a).

BRAF inhibition in A-375 was correlated to downregulation of the antiapoptotic Bcl-2-related protein Mcl-1 as well as to upregulation of the proapoptotic BH3 domain-only proteins Puma and Bim (24 kDa, isoform EL), as also shown previously.^10^ The finding that Puma was downregulated again after 48 h is explained by strongly induced apoptosis, which may lead to degradation of many proteins. Of the other apoptosis regulators investigated by Western blotting, antiapoptotic Bcl-2 and the proapoptotic transcription factor p53 were not regulated by TRAM-34 or vemurafenib. But antiapoptotic XIAP as well as proapoptotic Bax were downregulated at 48 h of combination treatment in course of massive apoptosis induction (Figure 3a). Thus Bcl-2 proteins appear of particular note for vemurafenib activity, but there was no indication that altered expression of Bcl-2 proteins was also critical for the combination effects seen here with TRAM-34.

Significant activation of the major effector caspase-3 upon combination treatment (24 and 48 h) was seen by its characteristic cleavage products of 17 and 15 kDa, suggesting a contribution of caspase-mediated pathways. Nevertheless, caspase activation appeared as not complete, seen by still high levels of procaspase-3 (Figure 3a). The role of caspases was further addressed by using the pancaspase inhibitor QVD-OPh. Pretreatment of A-375 with QVD-OPh (10 *μ*M) for 1 h resulted in significant but not complete reduction of apoptosis by vemurafenib alone (9%→3%) as well as by the combination (47%→13%). Similarly, cell viability was partly restored (16%→33% Figure 3b).

Proapoptotic and antiapoptotic Bcl-2 proteins control mitochondrial apoptosis pathways, for which loss of the MMP represents a critical step. Whereas in A-375, TRAM-34 and vemurafenib as single treatments remained without significant effect on MMP at 4 and 24 h, the combination resulted in a complete loss of MMP at 24 h (>90%), indicating full activation of mitochondrial apoptosis pathways at this time (Figure 3c). Similarly, 60% cells with low MMP were obtained in Mel-2a at 24 h. Only here, cells also showed response at 4 h (Figure 3d).

The decisive roles of Bcl-2 proteins and mitochondrial pathways were further confirmed by the results of Bcl-2 overexpression. Thus Vem/TRAM-induced apoptosis was completely prevented in a stably Bcl-2-overexpressing A-375 cell line (A375-Bcl-2), as compared with a mock-transfected cell clone (A375-pIRES; Figure 3e). Both cell clones had been previously established and characterized.^13^

**Figure 4:**
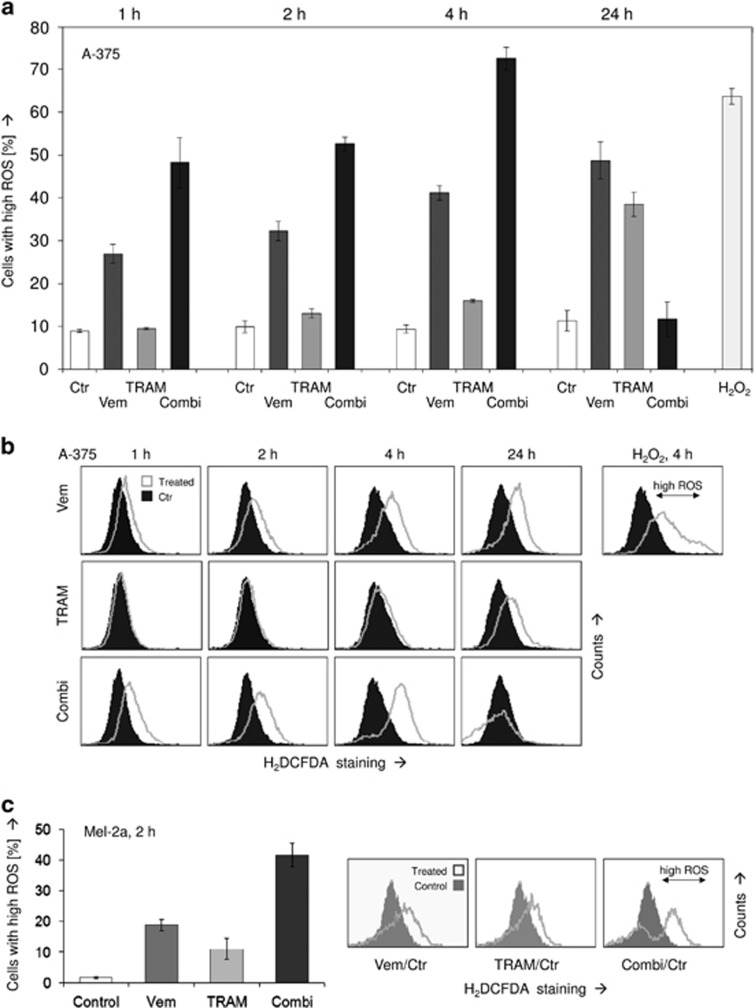
(**a**) Intracellular levels of ROS were determined in A-375 at 1, 2, 4 and 24 h of treatment with 30 *μ*M vemurafenib (Vem), 50 *μ*M TRAM-34 (TRAM) and the combination. The assay is based on staining with the ROS-sensitive dye H_2_DCFDA and subsequent flow cytometry. (**b**) Examples of treated cells *versus* controls are given. H_2_O_2_-treated cells served as positive control; cells with high ROS are indicated. The experiment was performed in triplicates; at 2 and 4 h, at least two independent experiments revealed highly comparable results. (**c**) Increased ROS levels are shown in Mel-2a at 2 h in response to vemurafenib (30 *μ*M), TRAM-34 and the combination. Examples are shown on the right side (triplicate determinations, two experiments)

Despite the obvious significance of lost MMP at 24 h, it should be considered at least in A-375 as a delayed effect, as less evident at 4 h (Figure 3c). Thus we looked for more initial effects. By this, we identified the generation of ROS as an immediate early event, already visible at 1 h of treatment. Thus ROS levels were consistently upregulated by vemurafenib single treatment raising from 27% of cells with high ROS at 1 h to 49% cells at 24 h. This effect further increased with the combination treatment resulting in 48% cells with high ROS at 1 h and 72% cells at 4 h (Figure 4a). Thus the whole cell population shifted toward higher ROS levels upon treatment (Figure 4b). The reproducible finding that ROS levels upon combination treatment were again downregulated at 24 h underlined the transient appearance of ROS, which was thus not a consequence of induced apoptosis. In parallel, we obtained ROS generation in Mel-2a by vemurafenib at 2 h, which was, with 19±2%, however, less than in vemurafenib-sensitive A-375. Nevertheless, upon combination treatment, ROS levels were raised up to 42±4% (Figure 4c).

**Figure 5:**
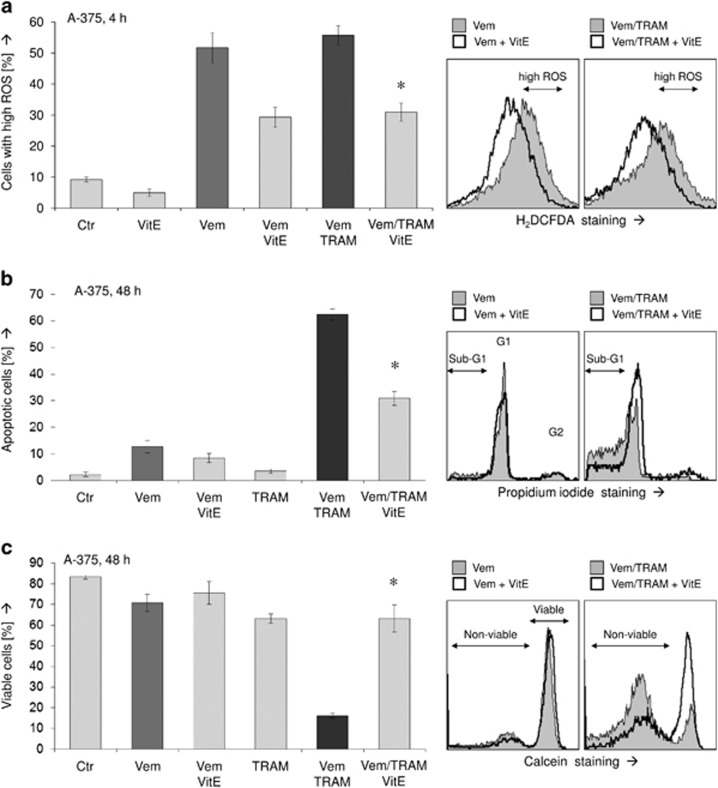
(**a**) Intracellular ROS levels were determined in A-375 at 4 h of treatment with 30 *μ*M vemurafenib (Vem), 50 *μ*M TRAM-34 (TRAM), the ROS scavenger vitamin E (VitE; *α*-tocopherol; 1 mM) and combinations. Examples on the right side demonstrate reduction of high ROS levels through VitE. (**b** and **c**) Induction of apoptosis (**b**) and loss of cell viability (**c**) were determined at 48 h in response to Vem (30 *μ*M), TRAM (50 *μ*M) and VitE (1 mM). All experiments were performed in triplicates, and at least two independent experiments showed highly comparable results; asterisks indicate statistical significance

**Figure 6:**
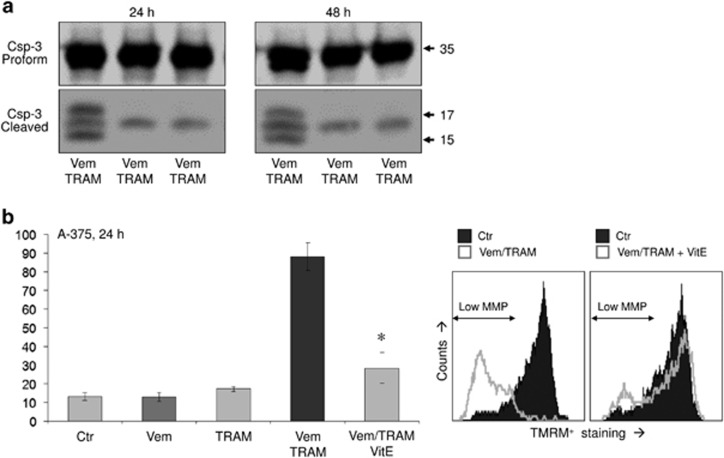
(**a**) Activated caspase-3 cleavage products seen in response to Vem/TRAM treatment at 24 and 48 h by Western blotting are abolished both by the pancaspase inhibitor QVD-OPh (10 *μ*M) and by VitE (1 mM). Two Western blottings with independent cell extracts revealed the same result. (**b**) Loss of MMP seen in A-375 at 24 h by Vem/TRAM treatment was significantly reduced after 1 h pretreatment with VitE. Experiments were performed in triplicates, and two independent experiments showed highly comparable results; asterisks indicate statistical significance

To prove the significance of ROS production, we performed experiments with the ROS scavenger *α*-tocopherol (VitE). In fact, pretreating A-375 cells for 1 h with 1 mM VitE reduced ROS levels upon vemurafenib and combination treatment by almost twofold (Figure 5a). This resulted in a concomitant twofold reduction of apoptosis (Figure 5b) and largely recovered cell viability (Figure 5c). Thus ROS produced by vemurafenib appeared as largely responsible for enhanced apoptosis upon combination treatment. Further demonstrating its signaling effect, caspase-3 activation by vemurafenib/TRAM was completely abolished by vitamin E, just as it was abolished by the pancaspase inhibitor QVD-OPh (Figure 6a). Also MMP was largely restored by vitamin E, locating also loss of MMP as downstream of ROS in the signaling cascade (Figure 6b).

## Discussion

The development of selective inhibitors for BRAF and MEK as well as immune checkpoint inhibitors such as anti-CTLA4 and anti-PD1 have revolutionized melanoma therapy in the past years leading to significant improvements for melanoma patients.^2, 3, 6, 7^ To further overcome therapy resistance and tumor relapse, combination therapies are of principle value as actually being demonstrated by combinations of BRAF and MEK inhibitors.^4, 5^ Also, combinations of targeted therapy and immune checkpoint inhibitors are considered.^24, 25^ Besides, many other combinations have been tested in melanoma experimental models. Enhanced efficiency was described for combinations of vemurafenib with different strategies, for example, with cyclin-dependent kinase 4 and 6 inhibitor,^26^ with inhibitors of epigenetic regulators^27^ or radiotherapy.^28^ Often toxicity is a limiting factor for *in vivo* use. Given a high number of possible targets in cancer cells, the repertoire of, in principle, suitable combination partners will grow tremendously in near future, giving much hope for further improvement of cancer therapies.

Inhibitors of membrane channels expressed in cancer cells represent additional, promising candidates that are still awaiting their full exploration. Potassium channels have critical roles in a wide variety of physiological processes. The group of Ca^2+^-dependent K^+^ channels contributes to cytoplasmic membrane hyperpolarization thus facilitating Ca^2+^ entry, a prerequisite for cell proliferation.^19^ The family member KCa3.1 is inhibited by clotrimazole, which itself is, however, not suitable for systemic application as hepatotoxicity results from non-specific effects on cytochrome P450. In contrast, the clotrimazole analogs TRAM-34 and ICA-17043 are more selective for KCa3.1 and lack P450-inhibitory activity.^21, 29^ Specificity of TRAM-34 was also proven in KCa3.1-negative HEK-293 cells, which did not respond, whereas responsiveness was recovered in KCa3.1-transfected cells.^23^

Knockout mice for KCa3.1 are viable and ICA-17043 was well tolerated in a clinical trial, showing almost no adverse effects.^29, 30^ Thus KCa3.1 may not be required for steady-state physiological balance but may be upregulated in disease situations. It may have critical roles in aberrant cell proliferation and cytokine synthesis in cancer and autoimmune disease.^29^ Blockade of KCa3.1 frequently results in G0/G1 cell cycle arrest and inhibitory effects on cytokine synthesis, which may be caused by suppressed calcium inflow.^31, 32^ KCa3.1 mRNA is overexpressed in different cancer cell lines, for example, of glioblastoma, pancreatic carcinoma, breast carcinoma and prostate carcinoma.^20, 29^ In a series of melanoma cell lines, mRNA and protein expression was shown, suggesting KCa3.1 expression as characteristic for melanoma.^23^ Also in nude mice, KCa3.1 inhibition by clotrimazole inhibited melanoma growth.^31^

Antitumor effects of KCa3.1 inhibition were correlated to suppression of cell proliferation and migration in breast cancer, endometrial carcinoma and hepatocellular carcinoma cells.^33, 34, 35^ In combinations, it increased the sensitivity of glioblastoma cells for radiotherapy^36^ and of glioma cells for temozolomide.^37^ In most instances, KCa3.1 blockers were cytostatic rather than cytotoxic or proapoptotic,^33, 38^ which may limit their use as monotherapy but may encourage for combination with proapoptotic agents. This was also seen in melanoma cells, namely, KCa3.1 inhibition by TRAM-34 decreased cell proliferation without directly affecting apoptosis, but it strongly sensitized melanoma cell lines for TRAIL-induced apoptosis.^23^

Here we prove TRAM-34 as a suitable combination partner for vemurafenib. Upon combination, apoptosis was strongly enhanced and even vemurafenib-resistant cells could be targeted. Cell viability and overall cell proliferation were completely abolished, whereas direct cytotoxc effects (cell lysis) remained at a low level. Concerning the proapoptotic mechanisms, activation of caspase-3, the mitochondrial pathway and elevation of ROS levels were shown. Enhanced apoptosis and loss of cell viability were evident not only for the standard concentration of 30 *μ*M vemurafenib but also for reduced concentrations, not indicating a threshold for mutual enhancement. Also, the combination effect appeared as representative for melanoma cells as seen in 3/3 BRAF-mutated melanoma cell lines.

Whereas caspase cascades and proapoptotic mitochondrial activation represent standard pathways in apoptosis control, the regulation of apoptosis by ROS is less understood. The significance of ROS for apoptosis induction has been proven by us previously,^17, 18^ and increased ROS levels in response to vemurafenib were also reported.^39, 40^ Here we prove ROS as a major factor in vemurafenib-induced apoptosis, in particular, in its combination with TRAM-34. This was shown by the ROS scavenger *α*-tocopherol, which strongly diminished apoptosis induction and partly restored cell viability. ROS was also upstream of other signaling steps, as it appeared already within 1 h, and *α*-tocopherol blocked both caspase activation and loss of MMP. As a possible origin of ROS, the endoplasmatic reticulum (ER) may be considered, as ER stress has already been linked to vemurafenib-induced apoptosis.^10^

In conclusion, these data shed more light on a frequently used but not completely understood melanoma therapy. ROS represents an initial and independent apoptosis pathway that is of particular importance for vemurafenib and its combination with TRAM-34 in melanoma cells. Better understanding may be helpful for further improving targeted therapy in melanoma.

## Materials and methods

### Cell culture

Three BRAF mutated human melanoma cell lines were used in the present study: A-375,^41^ Mel-HO^42^ and Mel-2a.^43^ A-375 subclones had been established previously by stable transfection of a pIRES-Bcl-2 plasmid for Bcl-2 overexpression (A375-Bcl-2) or by transfection of an empty pIRES plasmid (A375-pIRES, mock control).^13^ Cells were cultured at 37 °C, 5% CO_2_ with DMEM (4.5 g/l glucose; GIBCO, Invitrogen, Karlsruhe, Germany) supplemented with 10% FCS and antibiotics (Biochrom, Berlin, Germany). For analyses, 5 × 10^4^ and 2 × 10^5^ cells were seeded in 24- and 6-well plates, respectively.

The selective BRAF (V600E) inhibitor vemurafenib/PLX4032 (Selleck Chemicals, Houston, TX, USA) was used at a standard concentration of 30 *μ*M. This concentration theoretically corresponds to doses applied in patients (960 mg/12 h; MW=490 g/mol). In several experiments, also reduced concentrations (3 and 10 *μ*M) were included. TRAM-34, a selective inhibitor of the Ca^2+^-dependent potassium channel KCa3.1 (IK1), has been kindly provided by Dr. Heike Wulff, University of California, Department of Pharmacology, Davis, CA, USA.^21^ According to a previous study in melanoma cells,^23^ TRAM-34 was used at a standard concentration of 50 *μ*M. For caspase inhibition, the pan-caspase inhibitor QVD-OPh (Sigma-Aldrich, Taufkirchen, Germany) was used at 10 *μ*M, 1 h before other treatments started.

### Cell cycle analysis, apoptosis, cytotoxicity, cell viability, cell proliferation and adhesion

Quantification of apoptosis was performed by cell cycle analyses.^44^ Trypsinized cells were lysed in hypotonic buffer, and isolated nuclei were stained for 1 h with 40 mg/ml propidium iodide (Sigma-Aldrich). Cell fractions in G1, G2 and S-phase as well as sub-G1 cells were quantified by flow cytometry at FL3A with a FACS Calibur (BD Bioscience, Bedford, MA, USA). Owing to the washing out of small DNA fragments, nuclei with less DNA than in G1 (sub-G1) correspond to apoptotic cells with fragmented DNA.

Direct cytotoxicity (cell lysis) was determined by quantifying released LDH activity in cell supernatants by an LDH activity enzymatic assay (Roche Diagnostics, Penzberg, Germany), which was quantified in an ELISA reader later.

Cell viability was determined by staining cells with calcein-AM (PromoCell, Heidelberg, Germany), which is converted in viable cells to green-fluorescent calcein by intracellular esterases. Cells, grown and treated in 24-well plates, were trypsinized and stained with 2.5 *μ*g/ml calcein-AM at 37 °C for 1 h. Labeled cells were washed with PBS and measured by flow cytometry (FL2H).

For monitoring cell growth and attachment in real time, the xCELLigence system (OMNI Life Science, Bremen, Germany) was applied. Melanoma cells were seeded in special 96-well E-plates (50 000 cells per well), which have microelectrodes integrated in the bottom of the wells. Continuous measurement of electric resistance corresponds to attached cell numbers.

### MMP and ROS

MMP (Δψm) was determined by staining cells with the fluorescent dye TMRM^+^ (Sigma-Aldrich). Cells, grown and treated in 24-well plates, were harvested by trypsinization and stained for 20 min at 37 °C with 1 *μ*M TMRM^+^. After washing two times with PBS, cells were measured by flow cytometry (FL2H).

For determination of intracellular ROS, attached cells in 24-well plates were preincubated for 1 h with the fluorescent dye H_2_DCFDA (D-399, Thermo Fisher Scientific, Hennigsdorf, Germany, 10 *μ*M) before starting treatment with effectors. After treatment, cells were trypsinized, washed with PBS and analyzed by flow cytometry (FL1H). As positive control, cells were treated with H_2_O_2_ (1 mM, 1 h). For ROS scavenging, cells were pretreated for 1 h with 1 mM *α*-tocopherol (vitamin E, Fluka, Steinheim, Germany).

### Western blotting

For Western blotting, total protein extracts were obtained by cell lysis in 150 mM NaCl, 1 mM EDTA, 2 mM PMSF, 1 mM leupeptin, 1 mM pepstatin, 0.5% SDS, 0.5% NP-40 and 10 mM Tris-HCl, pH 7.5. Western blotting on nitrocellulose membranes was performed as described previously.^45^ Primary antibodies were: Cleaved caspase-3 (9664, rabbit, 1 : 1000, Cell Signaling, Danvers, MA, USA), caspase-3 proform (9662, rabbit, 1 : 1000, Cell Signaling), Mcl-1 (sc-12756, mouse, 1 : 200, Santa Cruz Biotech, Dallas, TX, USA), Bcl-2 (sc-492, rabbit; 1 : 200, Santa Cruz Biotech), Bax (sc-20067, mouse, 1 : 200, Santa Cruz Biotech), GAPDH (sc-32233, mouse, 1 : 1000, Santa Cruz Biotech), Puma (ab33906, rabbit, 1 : 1000, Abcam, Cambridge, UK), XIAP (no. 2042, rabbit, 1 : 1000, Cell Signaling), p53 (sc-126, rabbit, 1 : 500, Santa Cruz Biotech), ERK (no. 4695, rabbit, 1 : 1,000, Cell Signaling), pERK (no. 9101, rabbit, 1 : 1,000, Cell Signaling), and Bim (no. 559685, rabbit, 1 : 200, BD Biosciences, Heidelberg, Gemany). Secondary antibodies were: peroxidase-labeled goat anti-rabbit and goat anti-mouse (Dako, Hamburg, Germany; 1 : 5000).

### Statistical analyses

All assays were carried out in triplicate determinations and at least two independent experiments were performed. Also, Western blotting data were verified by at least two independent series of cellular extracts. Statistical significance was proven by Student's *t*-test (normal distribution), and *P*-values of <0.05 were considered as statistically significant.

## References

[bib1] Davies H, Bignell GR, Cox C, Stephens P, Edkins S, Clegg S et al. Mutations of the BRAF gene in human cancer. Nature 2002; 417: 949–954. 12068308 10.1038/nature00766

[bib2] Menzies AM, Long GV. Systemic treatment for BRAF-mutant melanoma: where do we go next? Lancet Oncol 2014; 15: e371–e381. 25079100 10.1016/S1470-2045(14)70072-5

[bib3] Garbe C, Peris K, Hauschild A, Saiag P, Middleton M, Bastholt L et al. Diagnosis and treatment of melanoma. European consensus-based interdisciplinary guideline - update 2016. Eur J Cancer 2016; 63: 201–217. 27367293 10.1016/j.ejca.2016.05.005

[bib4] Long GV, Weber JS, Infante JR, Kim KB, Daud A, Gonzalez R et al. Overall survival and durable responses in patients with BRAF V600-mutant metastatic melanoma receiving dabrafenib combined with trametinib. J Clin Oncol 2016; 34: 871–878. 26811525 10.1200/JCO.2015.62.9345

[bib5] Larkin J, Ascierto PA, Dréno B, Atkinson V, Liszkay G, Maio M et al. Combined vemurafenib and cobimetinib in BRAF-mutated melanoma. N Engl J Med 2014; 371: 1867–1876. 25265494 10.1056/NEJMoa1408868

[bib6] Hughes T, Klairmont M, Sharfman WH, Kaufman HL. Interleukin-2, ipilimumab, and anti-PD-1: clinical management and the evolving role of immunotherapy for the treatment of patients with metastatic melanoma. Cancer Biol Ther 2015; 29: PMID:26418961, Epub ahead of print. 10.1080/15384047.2015.1095401 PMC8726727 26418961

[bib7] Chen G, Davies MA. Targeted therapy resistance mechanisms and therapeutic implications in melanoma. Hematol Oncol Clin North Am 2014; 28: 523–536. 24880945 10.1016/j.hoc.2014.03.001

[bib8] Hanahan D, Weinberg RA. Hallmarks of cancer: the next generation. Cell 2011; 144: 646–674. 21376230 10.1016/j.cell.2011.02.013

[bib9] Eberle J, Kurbanov BM, Hossini AM, Trefzer U, Fecker LF. Overcoming apoptosis deficiency of melanoma-hope for new therapeutic approaches. Drug Resist Updat 2007; 10: 218–234. 18054518 10.1016/j.drup.2007.09.001

[bib10] Beck D, Niessner H, Smalley KS, Flaherty K, Paraiso KH, Busch C et al. Vemurafenib potently induces endoplasmic reticulum stress-mediated apoptosis in BRAFV600E melanoma cells. Sci Signal 2013; 6: ra7. 23362240 10.1126/scisignal.2003057 PMC3698985

[bib11] Berger A, Quast SA, Plötz M, Kuhn NF, Trefzer U, Eberle J. RAF inhibition overcomes resistance to TRAIL-induced apoptosis in melanoma cells. J Invest Dermatol 2014; 134: 430–440. 23955071 10.1038/jid.2013.347

[bib12] Pop C, Salvesen GS. Human caspases: activation, specificity, and regulation. J Biol Chem 2009; 284: 21777–21781. 19473994 10.1074/jbc.R800084200 PMC2755903

[bib13] Raisova M, Hossini AM, Eberle J, Riebeling C, Wieder T, Sturm I et al. The Bax/Bcl-2 ratio determines the susceptibility of human melanoma cells to CD95/Fas-mediated apoptosis. J Invest Dermatol 2001; 117: 333–340. 11511312 10.1046/j.0022-202x.2001.01409.x

[bib14] Plötz M, Eberle J. BH3-only proteins: possible proapoptotic triggers for melanoma therapy. Exp Dermatol 2014; 23: 375–378. 24673301 10.1111/exd.12399

[bib15] Fan TF, Wu TF, Bu LL, Ma SR, Li YC, Mao L et al. Dihydromyricetin promotes autophagy and apoptosis through ROS-STAT3 signaling in head and neck squamous cell carcinoma. Oncotarget 2016; 7: 59691–59703. 27474168 10.18632/oncotarget.10836 PMC5312341

[bib16] Dokic I, Mairani A, Niklas M, Zimmermann F, Chaudhri N, Krunic D et al. Next generation multi-scale biophysical characterization of high precision cancer particle radiotherapy using clinical proton, helium-, carbon- and oxygen ion beams. Oncotarget 2016; 7: 56676–56689. 27494855 10.18632/oncotarget.10996 PMC5302944

[bib17] Franke JC, Plötz M, Prokop A, Geilen CC, Schmalz HG, Eberle J. New caspase-independent but ROS-dependent apoptosis pathways are targeted in melanoma cells by an iron-containing cytosine analogue. Biochem Pharmacol 2010; 79: 575–586. 19799874 10.1016/j.bcp.2009.09.022

[bib18] Quast SA, Berger A, Eberle J. ROS-dependent phosphorylation of Bax by wortmannin sensitizes melanoma cells for TRAIL-induced apoptosis. Cell Death Dis 2013; 4: e839. 24113173 10.1038/cddis.2013.344 PMC3824654

[bib19] Wickenden A. K(+) channels as therapeutic drug targets. Pharmacol Ther 2002; 94: 157–182. 12191600 10.1016/s0163-7258(02)00201-2

[bib20] Schönherr R. Clinical relevance of ion channels for diagnosis and therapy of cancer. J Membr Biol 2005; 205: 175–184. 16362505 10.1007/s00232-005-0782-3

[bib21] Wulff H, Miller MJ, Hansel W, Grissmer S, Cahalan MD, Chandy KG. Design of a potent and selective inhibitor of the intermediate-conductance Ca2+-activated K+ channel, IKCa1: a potential immunosuppressant. Proc Natl Acad Sci USA 2000; 97: 8151–8156. 10884437 10.1073/pnas.97.14.8151 PMC16685

[bib22] Glaser N, Little C, Lo W, Cohen M, Tancredi D, Wulff H et al. Treatment with the KCa3.1 inhibitor TRAM-34 during diabetic ketoacidosis reduces inflammatory changes in the brain. Pediatr Diabetes 2016; doi: 10.1111/pedi.12396. (e-pub ahead of print). 10.1111/pedi.12396 27174668

[bib23] Quast SA, Berger A, Buttstädt N, Friebel K, Schönherr R, Eberle J. General Sensitization of melanoma cells for TRAIL-induced apoptosis by the potassium channel inhibitor TRAM-34 depends on release of SMAC. PLoS One 2012; 7: e39290. 22723988 10.1371/journal.pone.0039290 PMC3377761

[bib24] Prieto PA, Reuben A, Cooper ZA, Wargo JA. Targeted therapies combined with immune checkpoint therapy. Cancer J 2016; 22: 138–146. 27111910 10.1097/PPO.0000000000000182 PMC6478395

[bib25] Hassel JC, Lee SB, Meiss F, Meier F, Dimitrakopoulou-Strauss A, Jäger D et al. Vemurafenib and ipilimumab: a promising combination? Results of a case series. Oncoimmunology 2015; 5: e1101207. 27141385 10.1080/2162402X.2015.1101207 PMC4839308

[bib26] Tate SC, Burke TF, Hartman D, Kulanthaivel P, Beckmann RP, Cronier DM. Optimising the combination dosing strategy of abemaciclib and vemurafenib in BRAF-mutated melanoma xenograft tumours. Br J Cancer 2016; 114: 669–679. 26978007 10.1038/bjc.2016.40 PMC4800303

[bib27] Paoluzzi L, Hanniford D, Sokolova E, Osman I, Darvishian F, Wang J et al. BET and BRAF inhibitors act synergistically against BRAF-mutant melanoma. Cancer Med 2016; 5: 1183–1193. 27169980 10.1002/cam4.667 PMC4867668

[bib28] Zahnreich S, Mayer A, Loquai C, Grabbe S, Schmidberger H. Radiotherapy with BRAF inhibitor therapy for melanoma: progress and possibilities. Future Oncol 2016; 12: 95–106. 26616061 10.2217/fon.15.297

[bib29] Chou CC, Lunn CA, Murgolo NJ. KCa3.1: target and marker for cancer, autoimmune disorder and vascular inflammation? Expert Rev Mol Diagn 2008; 8: 179–187. 18366304 10.1586/14737159.8.2.179

[bib30] Ataga KI, Orringer EP, Styles L, Vichinsky EP, Swerdlow P, Davis GA et al. Dose-escalation study of ICA-17043 in patients with sickle cell disease. Pharmacotherapy 2006; 26: 1557–1564. 17064199 10.1592/phco.26.11.1557

[bib31] Benzaquen LR, Brugnara C, Byers HR, Gatton-Celli S, Halperin JA. Clotrimazole inhibits cell proliferation *in vitro* and *in vivo*. Nat Med 1995; 1: 534–540. 7585119 10.1038/nm0695-534

[bib32] Reich EP, Cui L, Yang L, Pugliese-Sivo C, Golovko A, Petro M et al. Blocking ion channel KCNN4 alleviates the symptoms of experimental autoimmune encephalomyelitis in mice. Eur J Immunol 2005; 35: 1027–1036. 15770697 10.1002/eji.200425954

[bib33] Zhang Y, Feng Y, Chen L, Zhu J. Effects of intermediate-conductance Ca(2+)-activated K(+) channels on human endometrial carcinoma cells. Cell Biochem Biophys 2015; 72: 515–525. 25608633 10.1007/s12013-014-0497-0

[bib34] Liu Y, Zhao L, Ma W, Cao X, Chen H, Feng D et al. The blockage of KCa3.1 channel inhibited proliferation, migration and promoted apoptosis of human hepatocellular carcinoma cells. J Cancer 2015; 6: 643–651. 26078795 10.7150/jca.11913 PMC4466414

[bib35] Zhang P, Yang X, Yin Q, Yi J, Shen W, Zhao L et al. Inhibition of SK4 potassium channels suppresses cell proliferation, migration and the epithelial-mesenchymal transition in triple-negative breast cancer cells. PLoS One 2016; 11: e0154471. 27124117 10.1371/journal.pone.0154471 PMC4849628

[bib36] Stegen B, Butz L, Klumpp L, Zips D, Dittmann K, Ruth P et al. Ca2+-activated IK K+ channel blockade radiosensitizes glioblastoma cells. Mol Cancer Res 2015; 13: 1283–1295. 26041939 10.1158/1541-7786.MCR-15-0075

[bib37] D'Alessandro G, Grimaldi A, Chece G, Porzia A, Esposito V, Santoro A et al. KCa3.1 channel inhibition sensitizes malignant gliomas to temozolomide treatment. Oncotarget 2016; 7: 30781–30796. 27096953 10.18632/oncotarget.8761 PMC5058717

[bib38] Shepherd MC, Duffy SM, Harris T, Cruse G, Schuliga M, Brightling CE et al. KCa3.1 Ca2+ activated K+ channels regulate human airway smooth muscle proliferation. Am J Respir Cell Mol Biol 2007; 37: 525–531. 17585114 10.1165/rcmb.2006-0358OC

[bib39] Corazao-Rozas P, Guerreschi P, Jendoubi M, André F, Jonneaux A, Scalbert C et al. Mitochondrial oxidative stress is the Achille's heel of melanoma cells resistant to Braf-mutant inhibitor. Oncotarget 2013; 4: 1986–1998. 24161908 10.18632/oncotarget.1420 PMC3875764

[bib40] Yu L, Gao LX, Ma XQ, Hu FX, Li CM, Lu Z. Involvement of superoxide and nitric oxide in BRAF(V600E) inhibitor PLX4032-induced growth inhibition of melanoma cells. Integr Biol (Camb) 2014; 6: 1211–1217. 25363644 10.1039/c4ib00170b

[bib41] Giard DJ, Aaronson SA, Todaro GJ, Arnstein P, Kersey JH, Dosik H et al. *In vitro* cultivation of human tumors: establishment of cell lines derived from a series of solid tumors. J Natl Cancer Inst 1973; 51: 1417–1423. 4357758 10.1093/jnci/51.5.1417

[bib42] Holzmann B, Lehmann JM, Ziegler-Heitbrock HW, Funke I, Riethmüller G, Johnson JP. Glycoprotein P3.58, associated with tumor progression in malignant melanoma, is a novel leukocyte activation antigen. Int J Cancer 1988; 41: 542–547. 3258589 10.1002/ijc.2910410412

[bib43] Brüggen J, Macher E, Sorg C. Expression of surface antigens and its relation to parameters of malignancy in human malignant melanoma. Cancer Immunol Immunother 1981; 10: 121–127.

[bib44] Riccardi C, Nicoletti I. Analysis of apoptosis by propidium iodide staining and flow cytometry. Nat Protoc 2006; 1: 1458–1461. 17406435 10.1038/nprot.2006.238

[bib45] Eberle J, Fecker LF, Hossini AM, Wieder T, Daniel PT, Orfanos CE et al. CD95/Fas signaling in human melanoma cells: conditional expression of CD95L/FasL overcomes the intrinsic apoptosis resistance of malignant melanoma and inhibits growth and progression of human melanoma xenotransplants. Oncogene 2003; 22: 9131–9141. 14668794 10.1038/sj.onc.1207228

